# Treatment outcomes of primary surgery versus chemoradiotherapy for T4 oropharyngeal cancers

**DOI:** 10.1097/MD.0000000000031987

**Published:** 2022-12-02

**Authors:** Ching-Heng Yen, Sheng-Yow Ho, Sung-Wei Lee, Chia-Chun Chen, Li-Tsun Shieh

**Affiliations:** a Department of Radiation Oncology, Chi Mei Medical Center, Liouying, Tainan, Taiwan; b Department of Radiation Oncology, Chi Mei Medical Center, Tainan, Taiwan; c Graduate Institute of Medical Science, Chang Jung Christian University, Tainan, Taiwan.

**Keywords:** oropharyngeal cancer, concurrent chemoradiotherapy, surgery

## Abstract

Concurrent chemoradiotherapy (CCRT) has been the standard of care for locally advanced diseases regardless of human papillomavirus infection status. Other treatment options include surgery followed by adjuvant therapy and induction chemotherapy followed by CCRT or radiotherapy. However, for locally advanced T4 laryngeal or hypopharyngeal diseases, surgery is preferred over CCRT. Given the improvement in the functional outcomes of surgery, examining the oncologic outcomes in OPSCC patients is critical. This study aimed to determine whether differences in overall survival (OS) exist between surgery and CCRT. Oropharyngeal cancer patients included in the cancer registry of our hospital from January 2014 to December 2018 were retrospectively analyzed. Patients with T4 disease who underwent curative treatment were identified. In this study, the primary and secondary outcomes were OS and disease-free survival (DFS), respectively. Potential confounding factors were also evaluated. Details regarding recurrence pattern were listed. From 2014 to 2018, 74 newly diagnosed oropharyngeal cancer patients were identified from our cancer registry database, 60 of whom satisfied our inclusion criteria. Our findings showed an OS of 25.5 months and DFS of 17.5 months. No significant difference in both of OS and DFS were observed between the surgery and CCRT cohorts. Sex, stage, second primary cancer, IC, and primary treatment were not correlated with DFS. Male sex was the only significant factor identified, with an HR of 0.2 for OS (95% confidence interval, 0.06–0.71). No significant difference in both OS and DFS were observed between the CCRT and surgery cohorts. CCRT remains the standard of care for locally advanced disease.

## 1. Introduction

Oropharyngeal squamous cell carcinoma (OPSCC) cases have rapidly increased throughout recent decades, with approximately 100,000 new cases annually worldwide.^[1]^ Despite the declining rates of OPSCC due to reduced smoking in developed countries, human papillomavirus (HPV) infections have increased incidence rates of the same.^[2]^ Concurrent chemoradiotherapy (CCRT) remains the standard of care for locally advanced disease regardless of HPV infection status. Other treatment options include surgery followed by adjuvant therapy and induction chemotherapy (IC) followed by CCRT or radiotherapy. Compared to surgery, CCRT promotes better organ preservation and lesser swallowing, social eating, and social contact problems.^[3]^ However, no randomized trials have compared treatment outcomes and side effects between surgical and nonsurgical approaches.

The current National Comprehensive Cancer Network treatment guidelines recommend CCRT as the standard of care for both laryngeal and hypopharyngeal cancers. However, for locally advanced T4 laryngeal or hypopharyngeal disease, surgery remains the preferred treatment strategy, despite the importance of organ preservation. Although over two thirds of patients with primary CCRT achieve laryngeal preservation, this compromises survival in T4 laryngeal cancer patients. Notably, the 3-year overall survival (OS) was only 40% in the CCRT group but 70% in the surgery group.^[4]^

Although high morbidity and poor functional outcomes following large resections have been a concern previously, operative techniques have advanced considerably. Surgical management with radial forearm free flap reconstruction in T4 oropharyngeal cancer can preserve oral diet function.^[5]^ Moreover, evidence has shown that minimally invasive transoral robotic simulation for OPSCC is less time-consuming, promotes better patient compliance, and has less complications compared to conventional surgery.^[6]^ Although it is a highly technical requirement, there are still many patients with T4 disease underwent transoral robotic simulation without sacrifice of oncologic outcome.^[7–9]^

Given the improved functional outcomes after surgery, examining the oncologic outcomes in OPSCC patients is critical. Considering the unclear data on survival after surgical intervention in those with T4 OPSCC, this study aimed to identify differences in OS between surgery and CCRT.

## 2. Methods

### 2.1. Participants

We retrospectively analyzed oropharyngeal cancer patients included in the cancer registry of our hospital from January 2014 to December 2018. Patients with T4 disease who underwent curative treatment were identified. The inclusion criteria were patients who were over 20 years old, had good performance status (ECOG not greater than 2), and received curative treatment. Exclusion criteria were no pathology report and cancer history before oropharyngeal cancer diagnosis. Age, sex, ECOG, stage, radiation dose, recurrence date, recurrence pattern, survival status, and the date of last follow-up were identified from the database. Follow-up started from the date of cancer diagnosis until December 2020.

### 2.2. Treatment

All patients underwent curative treatment. Surgery or definite CCRT was decided via tumor board discussions. Adjuvant therapy was based on the pathologic report. Patients with major risk factors, extranodal extension, or a positive margin were assigned to receive adjuvant CCRT. Patients with minor risk factors listed in the National Comprehensive Cancer Network guidelines, including pT3-4, pN2-3, nodal disease in level IV or V, perineural invasion, lymphovascular invasion, or close margin, underwent adjuvant radiotherapy or CCRT.

### 2.3. Outcomes

The primary outcome in this study was OS, defined as the duration from diagnosis to death or last follow-up. The secondary outcome was disease-free survival (DFS), defined as the duration from diagnosis to disease progression. Potential confounding factors evaluated included age, sex, stage, second primary cancer, complete arranged treatment, and IC. Details regarding recurrence pattern were listed.

### 2.4. Statistical analysis

We performed Kaplan–Meier analyses and assessed differences in OS and PFS using log-rank tests. Patients were censored upon death or last follow-up. Cox regression models were created for DFS after adjusting for age, sex, stage, second primary cancer, complete arranged treatment, and IC. We utilized 95% confidence intervals (95% CI) to the hazard ratios. *T* tests were used to evaluate differences in categorical variables. All statistical analyses were conducted using SPSS (version 24.0. Armonk, NY: IBM Corp), with a two-tailed *P* value of .05 indicating statistical significance. The research protocol was approved by the medical ethics committee of Chimei hospital. Given the retrospective nature of this study, informed consent was waived.

## 3. Results

From 2014 to 2018, 74 newly diagnosed oropharyngeal cancer patients were identified, among whom 60 satisfied our inclusion criteria. Table 1 summarized the demographic and clinical characteristics of the patients. The median age of the patients was 53.4 years old (ranging from 39 to 74). 95% (57 patients) of them were male and only 5% (3 patients) were female. 88% of patients had stage IVA disease and the others had stage IVB disease. 25% of them developed second primary cancer during follow-up. 42% patients experiences cancer recurrence. 28% patients had local recurrence, 12% had regional recurrence and 13% had distant metastasis. 38% patients received IC before the primary treatment. Personal habit, smoking, alcohol consumption and betel nuts chewing were found in 77%, 70%, and 90% of patients, respectively. Only 3 of the patients in the CCRT group did not received cisplatin-based concurrent treatment due to impaired renal function.

**Table 1:** Patient characteristics.

Characteristics	Patients (N = 60), n (%)
Age (yr), mean (range)	53.4 (39–74)
Disease-free survival (mo), median (range)	17.5 (1–81)
Overall survival (mo), median (range)	25.5 (3–81)
Sex	
Male	57 (95)
Female	3 (5)
Tumor stage	
IVA	53 (88)
IVB	7 (12)
Second primary cancer	15 (25)
Recurrence pattern	
Local	17 (28)
Regional lymph node	7 (12)
Distant metastasis	8 (13)
Total*	25 (42)
Induction chemotherapy	23 (38)
Primary treatment	
Surgery	41 (68)
Concurrent chemoradiotherapy	19 (32)
Chemotherapy regimen of concurrent treatment†	
Cisplatin	16 (84)
Cetuximab	2 (11)
Caroblantin	1 (5)
Smoking	46 (77)
Alcohol consumption	42 (70)
Betel nuts chewing	54 (90)

* Some of the patients experienced multiple recurrences.

† Only counts 19 patients who underwent CCRT as primary treatment.

CCRT = concurrent chemoradiotherapy.

Among the included patients, 41 and 19 underwent surgery and CCRT as primary treatment, respectively. After comparing the demographic and baseline clinical characteristics between the 2 cohorts (Table 2), no difference in sex, tumor stage, second primary cancer, smoking, alcohol consumption, betel nus chewing and recurrence site were observed. However, the CCRT cohort was older and had more patients receiving IC than the surgery cohort.

**Table 2:** Comparison of patients treated with surgery and CCRT.

Characteristics	Surgery (N = 41)	CCRT (N = 19)	*P* value
Age (yr), mean (range)	51 (39–67)	58 (43–74)	.004*
Sex			.95
Male	39 (95)	18 (95)	
Female	2 (5)	1 (5)	
Tumor stage			.50
IVA	37 (90)	16 (84)	
IVB	4 (10)	3 (16)	
Second primary cancer	11 (27)	4 (21)	.64
Recurrence pattern			
Local	11 (27)	6 (32)	.71
Regional lymph node	5 (12)	2 (11)	.85
Distant metastasis	5 (12)	3 (16)	.71
Induction chemotherapy	10 (24)	13 (68)	.001*
Smoking	32 (78)	14 (74)	.72
Alcohol consumption	31 (76)	11 (58)	.20
Betel nuts chewing	38 (93)	16 (84)	.32

CCRT = concurrent chemoradiotherapy.

**Figure 1.**
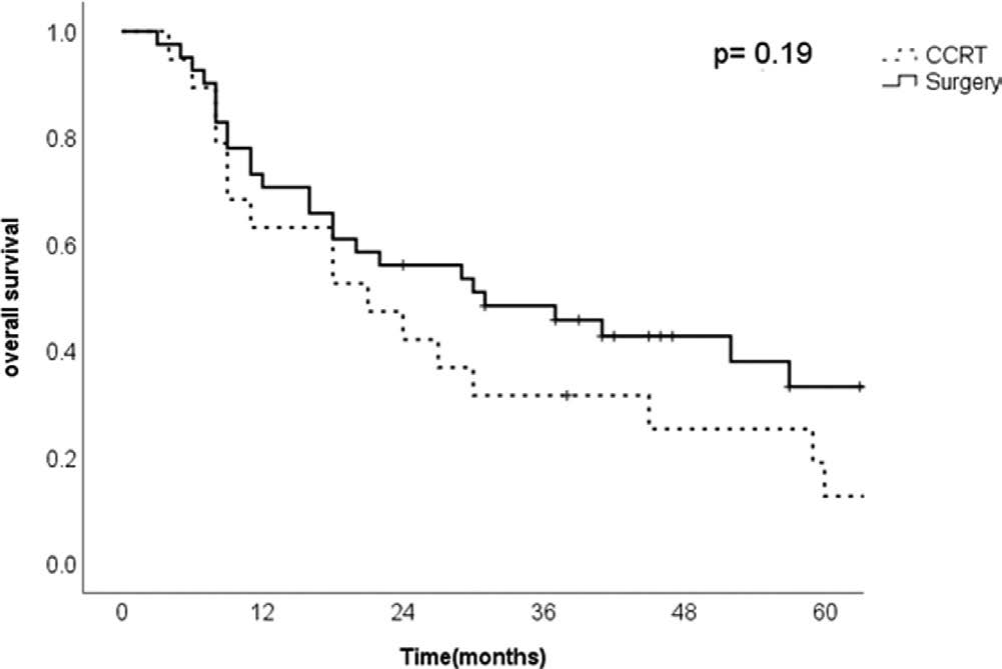
Overall survival between surgery and concurrent chemoradiotherapy.

**Figure 2.**
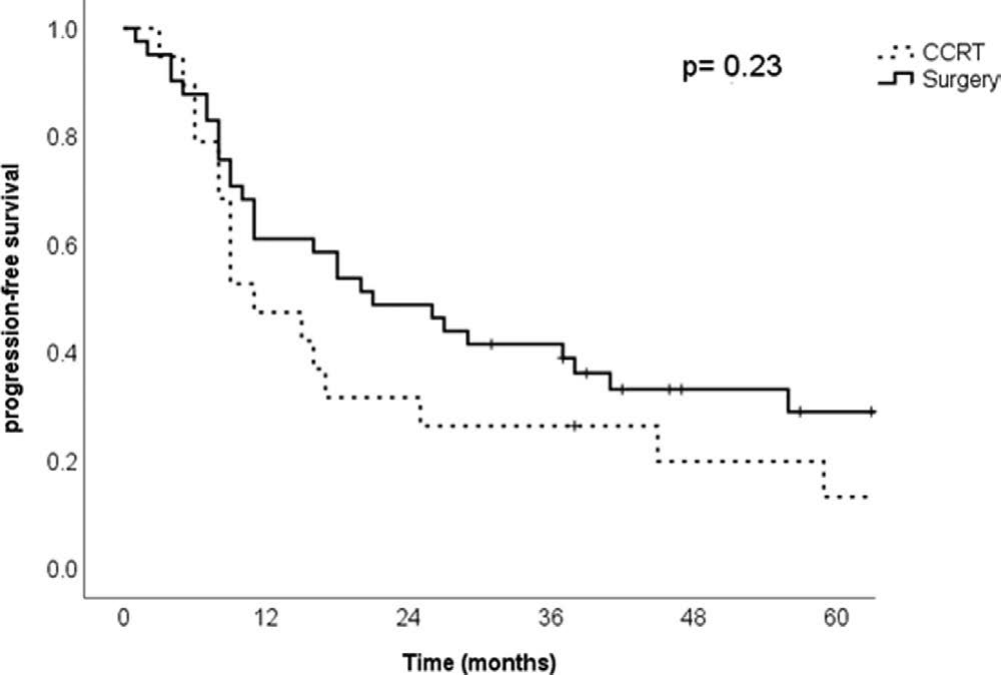
Disease-free survival between surgery and concurrent chemoradiotherapy.

OS and DFS were 25.5 and 17.5 months, respectively. The surgery and CCRT cohorts showed no significant differences in OS (*P* = .19) (Fig. 1). DFS was also not statistically different between the 2 cohorts (*P* = .23) (Fig. 2). The Cox regression model (Table 3) found that sex, stage, second primary cancer, IC, and primary treatment were not correlated with DFS. Male sex was the only significant factor, with an HR of 0.2 for OS (95% CI 0.06–0.71).

**Table 3:** Univariate Cox regression analyses of overall survival.

Variable	HR (95% CI)	*P* value
Age group (yr)		.16
<54	(Reference)	
≥54	1.55 (0.84–2.84)	
Sex		.01*
Female	(Reference)	
Male	0.2 (0.06–0.71)	
Stage		.41
IVA	(Reference)	
IVB	1.64 (0.50–5.34)	
Second primary cancer		.43
No	(Reference)	
Yes	1.30 (0.67–2.51)	
Induction chemotherapy		.68
No	(Reference)	
Yes	1.14 (0.61–2.14)	
Primary treatment		.20
CCRT	(Reference)	
Surgery	0.66 (0.35–1.24)	

CCRT = concurrent chemoradiotherapy.

## 4. Discussion

A National Cancer Data Base analysis reported that T4 laryngeal cancer patients undergoing total laryngectomy had superior OS compared to those undergoing CCRT.^[10]^ Several studies have suggested that T4 laryngeal cancer patients undergoing CCRT with organ preservation had inferior OS compared to those undergoing total laryngectomy.^[11–13]^ Total laryngectomies remains advantageous in patients with T4 disease. Organ preservation may only be a reasonable alternative in certain patients. Studies have compared treatment outcomes between surgery and CCRT in those with T4 hypopharyngeal cancer. Among patients with advanced hypopharyngeal cancer reported in the NCDB, primary surgery was associated with longer survival compared to primary CCRT after propensity score adjustment.^[14]^ The SEER database also confirmed the survival benefits of surgery such that CRT and surgery promoted a median survival of 20 and 25 months, respectively.^[15]^ Surgery is not the only preferred treatment for T4 oropharyngeal disease, unlike T4 hypopharyngeal and laryngeal diseases. Although a handful of studies have compared surgical and nonsurgical modalities for the treatment of stage III or IV OPSCC,^[16–18]^ their results have been conflicting. O’Connell et al demonstrated that among those with stage III to IV OPSCC, the surgery cohort had better disease-specific survival than did the CCRT cohort. However, a matched-pair analysis revealed that CCRT was as effective as surgery for treating advanced OPSCC. Moreover, only one retrospective study focused on T4 disease.^[16]^ Although, 131 patients from a single academic hospital showed that surgical treatment may be associated with improved outcomes, 75% of them were HPV-positive. Subgroup analysis showed that the survival benefits of surgery over CCRT were only present in the HPV-positive group and not in the HPV-negative cohort. In the current study, the surgery and CCRT cohorts had similar OS and DFS. Although the HPV status was not available in the current study, we believe that most of our patients were HPV-negative given the relatively low rate of HPV infection in patients with OPSCC in Taiwan.^[19–21]^ Thus, our results are consistent with those presented in the aforementioned study.

The Paradigm trial, which compared IC followed by CCRT and cisplatin-based CCRT alone in patients with locally advanced head and neck cancer,^[22]^ half of whom had oropharyngeal cancer, showed no difference in OS. The author concluded that the addition of IC could be an appropriate approach for advanced disease with high-risk for local or distant failure. Another study exploring the role of IC in patients with N2 or N3 head and neck disease showed that IC did not promote better OS compared to CCRT alone.^[23]^ They also concluded that IC cannot be recommended routinely in patients with N2 or N3 locally advanced head and neck disease. Overall, both studies failed to detect any survival benefits of IC followed by CCRT. Although IC followed by surgery had also been proposed, IC still remains unavoidable in clinical scenarios. A phase III trial by Zhang et al, which compared IC followed by surgery and postoperative radiotherapy and up-front surgery and postoperative radiotherapy in patients with locally advanced resectable oral squamous cell carcinoma, failed to demonstrate improved survival following TPF IC before surgery.^[24]^ TISOC-1 investigated the effectivity of a split-dose TPF IC before surgery for stage III/IVA oral and oropharyngeal cancer. The authors suggested that the trimodal treatment was well tolerated with good oncological results. However, IC still promoted no survival benefits in the mentioned study. For borderline resectable diseases, responders to IC had a lower probability of close or involved resection margin. For tumors extending close to vital organs, such as the spinal cord or brain stem, the radiation field would be better covered by the prescription dose in responders, which lowers the chances of marginal failure.

The current study has some limitations. First, several biases associated with the retrospective nature of the study were inevitable. Some crucial clinical factors, such as HPV status, chemotherapy regimen, response of induction therapy, and personal habits, were not available. Although incidence rates of HPV-positive OPSCC have been increasing across Taiwan, they still remain at around 30%, which is much less than that in Western countries.^[19–21,25]^ Moreover, most of the OPSCC cases in Taiwan were related to smoking, alcohol, and betel nuts, which can reduce the survival benefit in the HPV-positive group.^[19]^ Therefore, we believe that the missing data on HPV status would not alter our results. Second, the sample size was relatively small given our focus on T4 OPSCC. Third, treatment choice may have been subject to selection bias. Some patients who received CCRT were not candidates for surgery, regardless of whether the disease was unresectable or medically inoperable due to comorbidity. Lastly, data on side effects were unavailable.

In conclusion, no significant difference in OS and DFS were observed between the CCRT and surgery cohorts. Although surgery may have similar oncologic outcomes with CCRT, it is also important that patient almost need adjuvant RT or CCRT after surgery. Treatment burden may induce more side effects. Thus, surgery should only be considered in selected patient. CCRT remains the standard of care for locally advanced disease.

## Acknowledgments

The authors would like to thank department of cancer registry in Chi Mei hospital for providing research data.

## Author contributions

**Conceptualization:** Ching-Heng Yen, Li-Tsun Shieh.

**Data curation:** Sung-Wei Lee.

**Formal analysis:** Ching-Heng Yen.

**Funding acquisition:** Li-Tsun Shieh.

**Methodology:** Sheng-Yow Ho, Chia-Chun Chen.

**Resources:** Sheng-Yow Ho, Li-Tsun Shieh.

**Software:** Li-Tsun Shieh.

**Supervision:** Sheng-Yow Ho, Li-Tsun Shieh.

**Validation:** Sung-Wei Lee.

**Visualization:** Chia-Chun Chen.

**Writing – original draft:** Ching-Heng Yen.

**Writing – review & editing:** Li-Tsun Shieh.

## References

[1] Bray F Ferlay J Soerjomataram I. Global cancer statistics 2018: GLOBOCAN estimates of incidence and mortality worldwide for 36 cancers in 185 countries. CA Cancer J Clin. 2018;68:394–424. 30207593 10.3322/caac.21492

[2] Lechner M Liu J Masterson L. HPV-associated oropharyngeal cancer: epidemiology, molecular biology and clinical management. Nat Rev Clin Oncol. 2022;19:306–27. 35105976 10.1038/s41571-022-00603-7 PMC8805140

[3] Boscolo-Rizzo P Stellin M Fuson R. Long-term quality of life after treatment for locally advanced oropharyngeal carcinoma: surgery and postoperative radiotherapy versus concurrent chemoradiation. Oral Oncol. 2009;45:953–7. 19665919 10.1016/j.oraloncology.2009.06.005

[4] Vengalil S Giuliani ME Huang SH. Clinical outcomes in patients with T4 laryngeal cancer treated with primary radiotherapy versus primary laryngectomy. Head Neck. 2016;38(suppl 1):E2035–40. 26828197 10.1002/hed.24374

[5] Sims JR Moore EJ. Primary surgical management with radial forearm free flap reconstruction in T4 oropharyngeal cancer: complications and functional outcomes. Am J Otolaryngol. 2018;39:116–21. 29279248 10.1016/j.amjoto.2017.12.012

[6] Gangwani K Shetty L Seshagiri R. Comparison of TORS with conventional surgery for oropharyngeal carcinomas in T1-T4 lesions. Ann Maxillofac Surg. 2019;9:387–92. 31909020 10.4103/ams.ams_33_18 PMC6933975

[7] Yver CM Shimunov D Weinstein GS. Oncologic and survival outcomes for resectable locally-advanced HPV-related oropharyngeal cancer treated with transoral robotic surgery. Oral Oncol. 2021;118:105307. 33932874 10.1016/j.oraloncology.2021.105307

[8] Zevallos JP Mitra N Swisher–McClure S. Patterns of care and perioperative outcomes in transoral endoscopic surgery for oropharyngeal squamous cell carcinoma. Head Neck. 2016;38:402–9. 25351184 10.1002/hed.23909

[9] Weinstein GS O’Malley BW Jr Cohen MA. Transoral robotic surgery for advanced oropharyngeal carcinoma. Arch Otolaryngol Head Neck Surg. 2010;136:1079–85. 21079160 10.1001/archoto.2010.191

[10] Stokes WA Jones BL Bhatia S. A comparison of overall survival for patients with T4 larynx cancer treated with surgical versus organ-preservation approaches: a national cancer data base analysis. Cancer. 2017;123:600–8. 27727461 10.1002/cncr.30382

[11] Grover S Swisher-McClure S Mitra N. Total laryngectomy versus larynx preservation for T4a larynx cancer: patterns of care and survival outcomes. Int J Radiat Oncol Biol Phys. 2015;92:594–601. 26068492 10.1016/j.ijrobp.2015.03.004

[12] Dziegielewski PT O’Connell DA Klein M. Primary total laryngectomy versus organ preservation for T3/T4a laryngeal cancer: a population-based analysis of survival. J Otolaryngol Head Neck Surg. 2012;41(suppl 1):S56–64. 22569051

[13] Megwalu UC Sikora AG. Survival outcomes in advanced laryngeal cancer. JAMA Otolaryngol Head Neck Surg. 2014;140:855–60. 25144163 10.1001/jamaoto.2014.1671

[14] Hochfelder CG Mehta V Kabarriti R. Survival analysis of patients with advanced hypopharyngeal cancer comparing patients who received primary surgery to those who received chemoradiation: an analysis of the NCDB. Oral Oncol. 2021;121:105470. 34418696 10.1016/j.oraloncology.2021.105470 PMC8461571

[15] Hochfelder CG McGinn AP Mehta V. Treatment sequence and survival in locoregionally advanced hypopharyngeal cancer: a surveillance, epidemiology, and end results–based study. Laryngoscope. 2020;130:2611–21. 31821572 10.1002/lary.28452 PMC8214863

[16] Zenga J Wilson M Adkins DR. Treatment outcomes for T4 oropharyngeal squamous cell carcinoma. JAMA Otolaryngol Head Neck Surg. 2015;141:1118–27. 25902372 10.1001/jamaoto.2015.0764

[17] O’Connell D Seikaly H Murphy R. Primary surgery versus chemoradiotherapy for advanced oropharyngeal cancers: a longitudinal population study. J Otolaryngol Head Neck Surg. 2013;42:31. 23663568 10.1186/1916-0216-42-31 PMC3668157

[18] Kano S Homma A Hayashi R. Matched-pair analysis in patients with advanced oropharyngeal cancer: surgery versus concurrent chemoradiotherapy. Oncology. 2013;84:290–8. 23485940 10.1159/000346908

[19] Lorenzatti Hiles G Chang KP Bellile EL. Understanding the impact of high-risk human papillomavirus on oropharyngeal squamous cell carcinomas in Taiwan: a retrospective cohort study. PLoS One. 2021;16:e0250530. 33891627 10.1371/journal.pone.0250530 PMC8064583

[20] Hwang TZ Hsiao JR Tsai CR. Incidence trends of human papillomavirus-related head and neck cancer in Taiwan, 1995-2009. Int J Cancer. 2015;137:395–408. 25395239 10.1002/ijc.29330

[21] Wang CP Chen TC Hsu WL. Rising incidence of HPV positive oropharyngeal cancer in Taiwan between 1999 and 2014 where betel nut chewing is common. BMC Cancer. 2022;22:296. 35313837 10.1186/s12885-022-09407-5 PMC8939208

[22] Haddad R O’Neill A Rabinowits G. Induction chemotherapy followed by concurrent chemoradiotherapy (sequential chemoradiotherapy) versus concurrent chemoradiotherapy alone in locally advanced head and neck cancer (PARADIGM): a randomised phase 3 trial. Lancet Oncol. 2013;14:257–64. 23414589 10.1016/S1470-2045(13)70011-1

[23] Cohen EE Karrison TG Kocherginsky M. Phase III randomized trial of induction chemotherapy in patients with N2 or N3 locally advanced head and neck cancer. J Clin Oncol. 2014;32:2735–43. 25049329 10.1200/JCO.2013.54.6309 PMC4876357

[24] Zhong LP Zhang CP Ren GX. Randomized phase III trial of induction chemotherapy with docetaxel, cisplatin, and fluorouracil followed by surgery versus up-front surgery in locally advanced resectable oral squamous cell carcinoma. J Clin Oncol. 2013;31:744–51. 23129742 10.1200/JCO.2012.43.8820 PMC5569675

[25] Ho SY Kao WC Hsiao SY. Retrospective analysis of adjuvant radiotherapy in oral cavity or oropharyngeal cancer: feasibility of omitting lower-neck irradiation. PLoS One. 2022;17:e0266678. 35404969 10.1371/journal.pone.0266678 PMC9000126

